# Preparation of Antihypertensive Drugs in Biological Matrix with Solvent Front Position Extraction for LC–MS/MS Analysis

**DOI:** 10.3390/molecules27010205

**Published:** 2021-12-29

**Authors:** Kamila Jaglińska, Beata Polak, Anna Klimek-Turek, Robert Błaszczyk, Andrzej Wysokiński, Tadeusz Henryk Dzido

**Affiliations:** 1 Department of Physical Chemistry, Medical University of Lublin, Chodźki 4A, 20-093 Lublin, Poland; beata.polak@umlub.pl (B.P.); anna.klimek-turek@umlub.pl (A.K.-T.); tadeusz.dzido@umlub.pl (T.H.D.); 2 Department of Cardiology, Medical University of Lublin, Jaczewskiego 8, 20-090 Lublin, Poland; robert.blaszczyk@umlub.pl (R.B.); andrzej.wysokinski@umlub.pl (A.W.)

**Keywords:** sample preparation procedure, thin-layer chromatography, solvent front position extraction, LC–MS/MS, bovine serum albumin (BSA), antihypertensive drugs

## Abstract

Solvent front position extraction procedure was used to prepare biological samples containing selected antihypertensive drugs (ramipril, lercanidipine, indapamide, valsartan, hydrochlorothiazide, perindopril, and nebivolol). Substances separated from the biological matrix components (bovine serum albumin) were quantified by means of liquid chromatography coupled with tandem mass spectrometry (LC-MS/MS). Sample preparation process was performed with the use of a prototype horizontal chamber with a moving pipette driven by a 3D printer mechanism enabling a controlled eluent flow velocity. Application of this device was advantageous for simultaneous preparation of several samples for further quantitative analysis, with a synchronized reduction of manual operations and solvent consumption. Quantitative results obtained for the majority of the investigated antihypertensive drugs in a complex biological matrix were satisfactory. The values of the %RSD were around 5% for six of the seven substances (with the exception of indapamide). The method exhibits a suitable accuracy (the relative error percentage was below 10% for most drugs). The values of LOD and LOQ were in the range of 1.19 µg/L–8.53 µg/L and 3.61 µg/L–25.8 µg/L, respectively.

## 1. Introduction

Isolation of drugs and/or their metabolites from the biological matrix is a real challenge for the analyst. Blood, urine, or saliva are the main sources of such material. Due to the presence of additional compounds (salts, acids, bases, proteins, or other organic components), often similar to the substances of interest, the obtained sample is of a very complex nature. Hence, the primary step of sample preparation is to purify it and selectively isolate the target analytes from other biological matrix components. The latter process is the basic step for further quantification of the investigated analytes by means of the HPLC, MS, and LC–MS techniques [1,2].

According to statistics, cardiovascular diseases are the leading cause of death [3,4] among patients nowadays. Treatment of CV diseases requires application of combinations of drugs with different pharmacological activities and different mechanisms of action. Several antihypertensive agents, e.g., angiotensin-converting enzyme inhibitors, calcium channel blockers, angiotensin II receptor blockers, and beta-blockers, are often used to treat CV diseases. However, hypotensives are also capable of reducing the risk of cardiovascular complications. Generally, hypertensives have two mechanisms of action: they inhibit or reduce the vascular wall contraction, they can also reduce body fluid volumes (including blood).

Thus, in order to prevent severe cardiovascular problems, it is essential that the treatment should be based on monitoring the blood concentration of the used therapeutic agent. This will ensure optimizing the drug dosage in each individual patient [5]. In the light of this, it is necessary that appropriate preparation of biological samples containing the above-mentioned compounds should precede determination of blood concentration of the applied therapeutic agent

There are many methods for isolating antihypertensive drugs and their metabolites from the biological matrix. Solid-phase extraction (SPE) [6,7,8] and precipitation [9,10,11] are some of the most popular ones, and, according to some reports, they are often combined with evaporation and reconstitution of the sample [11,12,13]. D. Liu et al. used solid-phase extraction to isolate hydrochlorothiazide from a clinical sample of the human plasma [12]. For this purpose, a Quadra 3 SPE semi-automatic filtration system with Waters Oasis MCX SPE columns was used. The extraction of the analyte from the biological matrix was carried out in several stages at the same time and required a large consumption of a few solvents. An additional disadvantage of the method was the need to evaporate the eluate in a nitrogen stream. Considering the above, the main SPE disadvantages are the possibility of a partial loss of the analyte due to incomplete desorption, consumption of large volumes of solvents, and selection of the correct and expensive columns.

In 2016, De Nicolo et al. used precipitation in the preparation of a biological sample containing ten antihypertensive drugs [14]. The process included protein denaturation, vortexing, centrifugation, supernatant evaporation, dry extract dissolving in an appropriate solution, and finally, vortexing again. This process is time-consuming and requires the use of an expensive laboratory centrifuge with a temperature control function. All this indicates that precipitation is a quite troublesome and expensive method of preparation of biological samples containing antihypertensive drugs, also difficult to use in routine tests.

The classification of SPE and protein precipitation techniques based on the most important analytical parameters are presented in Table 1.

According to the literature, thin-layer chromatography/high-performance thin-layer chromatography (TLC/HPTLC) has been successfully applied to isolate and perform quantitative analysis of a single component within a mixture [15]. Such a procedure requires the connection of the TLC or HPTLC technique with densitometry. The advantage of this approach may be that the sample preparation step before the quantitative analysis is not required because the plate is used only once. However, a relatively high LOD and LOQ threshold and lower reproducibility compared to the HPLC or LCMS technique is some disadvantage of such a procedure [15]. Nevertheless, applying the TLC/HPTLC technique may be attractive when used in the sample purification stage before their quantitation with the use of instrumental analysis [16].

Solvent front position extraction (SFPE), developed by our Department, is an alternative to the methods mentioned above. The SFPE procedure is based on isolation of the investigated substances from the matrix (e.g., albumin) using an adsorbent layer and forming a single spot/zone of the substances and internal standard at the solvent front position of a chromatographic plate. The new prototype device, equipped with a 3D printer’s mechanism, delivers the mobile phase at a controlled velocity onto the adsorbent layer by a pipette moving slightly above the chromatography plate. The pipette can move on three axes at different speeds, deliver the mobile phase anywhere on the chromatography plate and develop chromatograms in any direction [17]. Thus, using the 3D printer’s mechanism makes the method semiautomatic. During the procedure, the samples were applied as droplets onto the chromatographic plate surface using an automatic pipette. Such a procedure for sample application is convenient because it does not require sophisticated equipment and can also be applied outside the laboratory. Since a semiautomatic pipette delivers the solvent with a controlled velocity, its consumption is lower than with SPE.

In addition, since the sample is applied onto an adsorbent thin-layer of the chromatographic plate, the consumption of the adsorbent is lower compared to the SPE, hence, it lowers the cost of such a procedure. What is more, compared to the precipitation method, in SFPE, the centrifugation step may be skipped. All the advantages mentioned above are the SFPE benefits [17].

In our previous article, we focused only on the effect of albumin sorbent impregnation hen’s (egg white and bovine serum albumin) on the drug sample preparation process [18]. It turned out that isolation of substances from a biological matrix is more complicated and requires additional development of chromatograms to obtain the investigated substances in the front position of the mobile phase [18]. Hence, the purpose of this paper is to check the suitability of SFPE for the preparation of biological samples with antihypertensive drugs before their further quantitative determinations. This article presents preliminary results in this field. In our work, we focused on the isolation of the following medicines: ramipril, perindopril (class of angiotensin-converting enzyme inhibitor), lercanidipine (calcium channel blocker), indapamide, hydrochlorothiazide (diuretics), valsartan, telmisartan (angiotensin II receptor blockers), and nebivolol (beta-blocker). Their combination is used in the treatment of cardiovascular problems [19,20].

## 2. Results and Discussion

### 2.1. Preliminary Research—SFPE Procedure Optimization

According to the SFPE procedure postulate, the substance (or substances) and the standard internal zones on the chromatographic plate should migrate to the distance which allows the coefficient ^n^R_f_ value (relative distance migration of a solute after the n-th development) equal to at least 0.99 [17,21] to be obtained. Preliminary research was performed to find a chromatographic system that fulfills this postulate. Therefore, to reach this requirement, the number of chromatogram developments must be determined before applying the SFPE procedure of sample preparation. The following adsorbents were used in the initial experiments: silica gel, sorbents with C18, CN, diol, and cellulose. The subsequent mobile phases were tested: acetone, acetonitrile, ethyl acetate, isopropanol, toluene, methanol. The influence of pH on solute retention was also examined.

According to the preliminary results for methanolic samples of the investigated compounds, the best stationary phase for the first and second groups of substances was silica gel, while for the third group development, it was diol sorbent (Table 2).

Regarding mobile phase choice, methanol was the most appropriate for the II sample of substances (valsartan, hydrochlorothiazide, telmisartan) and III sample of substances (ramipril, nebivolol, perindopril). It turned out that only one development of the chromatogram allowed the retardation factor R_f_ > 0.9 to be reached for all substances from the above-mentioned groups. So, the minimum number of developments that guarantees the values ^n^R_f_ higher or equal to 0.99 is two. This outcome meets the SFPE postulate. For ramipril, lercanidipine, indapamide, perindopril (sample I) 0.1% formic acid in methanol was used as the mobile phase (Table 3). The ^n^R_f_ ≥ 0.99 was obtained after the second development of the chromatogram (the same results were obtained for 0.1% ammonia in methanol except for perindopril (Table 3); therefore, this mobile phase was omitted in further studies).

In the SFPE procedure optimization step, the chromatographic plates were impregnated with a bovine serum albumin solution to determine the effect of the albumin solution on the retention factor of the investigated substances. We believed that the obtained results may give valuable data on, among others, the degree of binding of selected drugs by albumins. Moreover, it should allow the application of an appropriate procedure to prepare biological samples using the SFPE technique coupled with LC–MS/MS for solute determination. The experiments performed with albumin impregnated sorbent for all samples were carried out using the same mobile phase as in the systems without impregnation. The obtained results showed that the presence of albumin on the sorbent surface caused a solute retention increase. It provided evidence for the drug–protein interactions [22,23,24]. Additionally, a change in the spot color (from deep blue to light blue) of the telmisartan was observed. This was the effect of binding this drug to albumin [22,23,24].

Based on the results obtained from the impregnated systems, the SFPE procedure was modified for the preparation of the biological samples (by the addition of bovine serum albumin). Such samples were found to require additional development of the chromatogram to make the spot reach the eluent front position, compared to the methanolic ones.

### 2.2. Quantization Result Procedure

Before instrumental analysis, the quantitative recovery of the substance from the matrix was investigated. At this stage of the research, the effectiveness of the SFPE procedure of biological sample preparation for LC–MS/MS analysis was checked. Hence, analyte-standard concentration ratios in the basic sample (without sample preparation) and SFPE as the preparation technique were compared. In undertaking this, a different internal standard was used for each group of the analyte mixture (details in Section 3.2). Such a procedure was used to eliminate errors related to the possible diminishing of the substance acting as a standard. This effect may come about when a multistage sample preparation procedure is applied or when the analytes are part of a rich biological matrix (e.g., with albumin).

#### 2.2.1. Methanolic Sample

**Figure 1:**
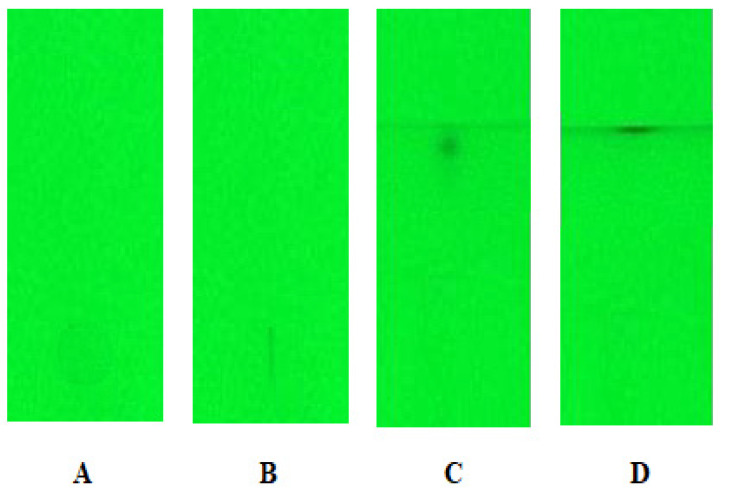
The mixture (methanolic sample) chromatograms for the first group of substances: (**A**) after sample application; (**B**) after starting spot narrowing; (**C**) after first development; (**D**) after second development. Stationary phase: HPTLC silica gel 60 F_254_. (Merck), mobile phase: 0.1% formic acid in methanol.

Regarding the examining of the methanolic solutions of the investigated solutes, samples were applied as a droplet onto the adsorbent layer of the chromatographic plate using the automatic pipette. This method of application does not require the use of advanced equipment. What is more, it is fast, convenient, and can be performed outside the laboratory. Additionally, considering biological samples containing albumins, due to their large viscosity and impurities, such a method of application is an alternative to the automatic applicator. After this step, the chromatograms were developed using the SP2 variant of the procedure (details in Section 3.6.2). The actions of the SFPE procedure for the first group of substances are shown in Figure 1 (chromatograms of one track are shown).

The use of the prototype horizontal chamber with a 3D printer mechanism with a controlled eluent flow allowed for the simultaneous preparation of several samples for further quantitative analysis. This process requires minimal involvement in manual operations. The advantage of the use of the device mentioned above is the possibility of choosing the place on the chromatographic plate surface to deliver the mobile phase and the direction of its development. Additionally, the semiautomatic procedure of the solute start zones narrowing is less time-consuming compared to the “manual” narrowing presented in our previous article [17].

After applying the SP2 procedure, the solutes were extracted (isolated) from the solvent front position of the chromatographic adsorbent layer with methanol comprising 0.1% formic acid using the CAMAG TLC–MS interface. The representative results for all (I-III) groups of substances are presented in Table 4.

Quantitative determination of all solutes was at a satisfactory level. %RSD and %RE of the results obtained by the direct (dLC–MS) procedure and by the LC–MS/MS combined with the SFPE technique did not exceed 5%. Therefore the method is precise and characterized by high accuracy. Compound recovery at the level of 100% and low detection and quantification limits additionally confirm the effectiveness of the method for methanol samples.

#### 2.2.2. Serum Albumin Samples

**Figure 2:**
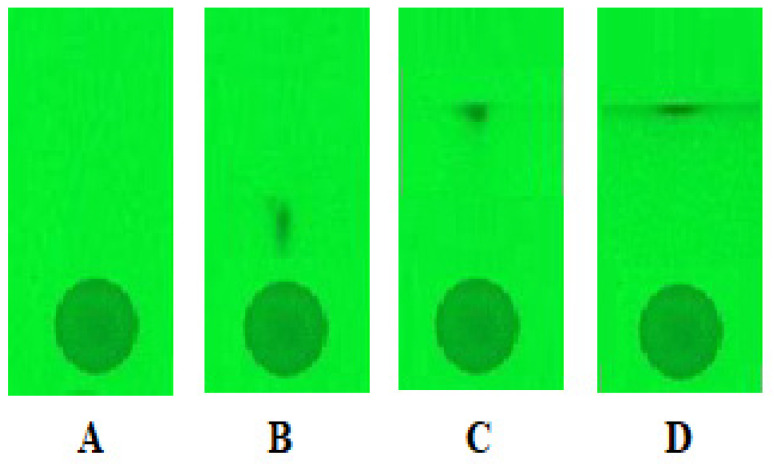
The mixture(bovine serum albumin sample) chromatograms for the first group of substances: (**A**) after sample application; (**B**)after wetting the spot twice and developing the chromatogram for the first time; (**C**) after second development; (**D**) after third development. Stationary phase: HPTLC silica gel 60 F_254_ (Merck), mobile phase: 0.1% formic acid in methanol.

In the next step, the prototype device for the SFPE procedure was applied for isolation of selected antihypertensive drugs from the biological matrix (bovine serum albumin). The chromatograms were developed using the P3 mode (details in Section 3.6.2). Figure 2 shows the steps of the P3 procedure for the bovine serum albumin sample containing the first group substances (chromatograms of one track are shown).

Procedure P3 was the same for the II and III groups of solutes. The starting spots were not narrowed, as compared to the methanolic samples. Considering samples with a biological matrix, a so-called “funnel” effect (Figure 2B) was formed during the development of the chromatograms [25]. This results in a narrowed zone of the substance. The solutes from the developed chromatogram (Figure 2D) were extracted from the solvent front position with methanol containing 0.1% formic acid using the CAMAG TLC–MS Interface. The results obtained for all groups of substances are presented in Table 5 (all data calculated in the same way as for methanolic samples, details in Table 4**.**

The %RSD value on the verge of error was 5% for six of the seven substances (the exception is indapamide). This is acceptable and shows that the prototype equipment used to isolate the investigated substances gives reproducible results. The percentage difference of the results obtained by the direct (dLC–MS) and the LC–MS/MS combined with the SFPE procedure is lower than 5% only for valsartan and perindopril (recovery of about 100%). For the other investigated drugs, the relative error (%RE) range was 20% (lercanidipine, ramipril, hydrochlorothiazide, nebivolol), except for indapamide. This was due to the strong binding of these solutes by serum proteins, which led to their elution from the matrix [22] being hindered. Hence, to improve the isolation, some strong solvent should be added. Thus, the effect of acetonitrile presence in the sample on the degree of solute isolation from the matrix components (i.e., bovine albumin) was checked. It was verified using the SFPE procedure.

In this procedure, the suspension of a mixture consisting of the sample and acetonitrile was applied on the chromatographic plate (details in Section 3.2). The following steps were identical to those with the bovine serum albumin samples (variant P3 of the SFPE procedure). The obtained results from this verified procedure are presented in Table 5. Application of the new approach showed that results for six of the seven investigated compounds were very satisfactory in terms of relative error (%RE) and solute recovery (%Recovery). The indapamide was the exception to these observations. The reason for such behavior will be explained in the future. The percentage differences of the results obtained by the (direct) dLC–MS and by the LC–MS/MS combined with the SFPE procedure were lower than the value obtained for biological samples without the addition of acetonitrile. In the case of lercanidipine, ramipril, and nebivolol, the relative error was below 10%, while for hydrochlorothiazide, it decreased from 20.57% to 13.28% (Table 5). Application of our technique significantly improved recovery of the substance (the values of recovery were in the range of 86.72–102.39%, respectively), which, additionally, demonstrates the high accuracy of this approach. The LOD and LOQ values were comparable to the previously discussed procedures.

Thus, application of the procedure with acetonitrile improved extraction of the studied drugs from the biological matrix. This outcome probably came about due to protein precipitation [26,27]. The presence of acetonitrile changes the structure of the BSA molecule [26,27]. It diminishes the degree of drug–protein binding. Despite the precipitation procedure, the sample still contained the matrix components, which might have affected the quantitative analysis. Therefore, in order to isolate the investigated solutes from undesirable impurities, it was necessary to use the SFPE approach. 

#### 2.2.3. SFPE Procedure Analysis

Since the timing of analysis and solvent consumption is crucial for analytical methods in the next step of our consideration, we decided to determine these parameters. They were calculated for a single bovine serum albumin sample prepared using the SFPE procedure. The time was measured from the moment of the sample application onto a chromatographic plate (5 × 10 cm) until the analyte extraction from the sorbent layer into the vial. In turn, the solvent consumption was then calculated by summing up the volumes used to wet the spots, develop the chromatograms and extract the investigated solutes into the vials. The obtained values were then divided by the number of the applied spots (details in Section 3.6), thus obtaining the real analysis time and amount of solvent needed to prepare one biological sample. The results are presented in Table 6. The preparation time of one biological sample was about 8 min, while the amount of the used solvent was 0.41 mL.

The SFPE procedure can also be performed on a 10 × 20 cm chromatography plate. With such a plate, it is possible to apply 32 spots, 16 on each side of the plate, and develop chromatograms in two directions. With this assumption, calculated theoretical values (Table 6), i.e., the time to prepare a single sample (2.40 min) and the required amount of solvent (0.38 mL) in the case of a plate twice as large were derived. Switching to a chromatographic plate twice as large and developing the chromatograms in two directions was found to reduce the sample preparation time by almost four times, as well as to reduce solvent consumption. This confirms that the SFPE procedure as a technique for the preparation of biological samples containing selected antihypertensive drugs is fast and economical.

#### 2.2.4. Comparison of Procedures

This section compares the main analytical aspects characterizing our procedure with the analytical features of the contemporary methods used, i.e., SPE and precipitation. For this purpose, the selected analytical parameters of the SFPE are summarized in Table 7 and then compared with the data in Table 1.

As reported in the literature, precipitation of proteins and extraction of metabolites from the biological matrix require the use of a solvent volume three times larger than the plasma volume (0.2–0.5 mL) [25]. The smallest volume of the matrix requires, approximately 0.6 mL of a solvent in order to prepare one sample, while, extraction of analytes from biological fluids by means of SPE extraction requires consumption of solvent in the range of 2.5–8 mL per sample (Table 1). With these values in mind and due to much lower solvent consumption, our extraction technique prevails significantly over precipitation and solid-phase extraction. It is a very desirable feature, in line with the green chemistry concept, where efforts are made to minimize the solvent volume [28]. Less solvent consumption also means lower analysis costs. The great advantage of our technique is the short sample preparation time (about 2.4 min per sample). Compared to the time of sample preparation with classical methods, it is usually comparable (in the case of SPE, it ranges from 1.5–3 min (Table 1)); however, it does happen that in some cases, it is sometimes lower (in precipitation, it even reaches 5 min—details in Table 1). Our procedure is the least complicated when comparing the number of steps (number of operations: six). Additionally, there is no need to use expensive SPE columns, which are used only disposable. What is more, there is no need to cool and centrifuge the sample, compared to precipitation.

The significant advantage of our approach is that the sample with the matrix is applied onto the chromatographic plate. Last but not least, the procedure carried out in this way is very easy to fully automate.

In summary, our extraction procedure is an alternative to classic extraction methods, mainly due to the much lower solvent consumption.

## 3. Materials and Methods

### 3.1. Materials and Reagents

Chromatographic glass plates, HPTLC silica gel 60 F_254_, HPTLC silica gel 60 RP-18 F_254_, HPTLC diol F_254_, HPTLC CN F_254_, and TLC cellulose were obtained from Merck (Darmstadt, Germany). Methanol, toluene, ethyl acetate, acetone, isopropyl, and acetonitrile of analytical grade were from Avantor (Gliwice, Poland). Deionized water was produced in the laboratory with the demineralizer HLP 5 from Hydrolab (Straszyn, Poland). The reagents used to prepare the acidic and basic mobile phases were as follows: formic acid (99.5%) and ammonia (25%) were also purchased from Avantor (Gliwice, Poland). Methanol and formic acid LC–MS grade were purchased from Merck (Darmstadt, Germany). Antihypertensive drugs (hydrochlorothiazide, indapamide, lercanidipine, nebivolol, telmisartan, valsartan) were donated from the Cardiology Clinic of the Independent Public Clinical Hospital No. 4 in Lublin (Lublin, Poland). Perindopril and ramipril were bought from Sigma–Aldrich (St. Louis, MO, USA). Albumin bovine fraction V (BSA) was from NzyTech–Genes & Enzymes (Lisbon, Portugal).

### 3.2. Preparation of Internal Standards and Analyte Solutions

Stock solutions of the tested antihypertensive drugs (hydrochlorothiazide, indapamide, lercanidipine, nebivolol, telmisartan, valsartan) were prepared by extracting the active substances from tablets. For each drug, an appropriate number of pills was weighed and then ground in a mortar. Then, the powder was weighed and transferred to 50 mL volumetric flasks. Next, 30 mL of the extracting solvent (methanol) was added. The methanol-drug suspensions were sonicated for 20 minutes with the use of ultrasonic power (EMAG, Juszczyn, Poland). After this time, the solvent was added to obtain a final volume of 50 mL. The mixtures were subsequently filtered through quantitative soft filters with a diameter of 150 mm (AlfaChem, Poznań, Poland). In the case of perindopril and ramipril, the solutions were prepared by dissolving appropriate amounts of standard substances in methanol. The concentrations of the standard solutions of all substances were equal to 1 mg/mL.

Afterwards, standard solutions were mixed in appropriate proportions to obtain the final test mixture solutions. Mixture I was prepared by mixing ramipril, lercanidipine, indapamide, and perindopril as the internal standard. Mixture II consisted of valsartan, hydrochlorothiazide, and telmisartan as the internal standard, while mixture III comprised perindopril, nebivolol, and ramipril as the internal standard. Each of the solutions was appropriately diluted. Concentrations of standards in mixture I were equal to 2.5 µg/mL for each component. The content of the drug in mixtures II and III was equal to 3.3 µg /mL. In the final stage the volumes of 10 µL of the previously prepared mixture I, II, or III were filled up to 1 mL with methanol or bovine serum albumin solution (0.04 g/mL in water).

Simultaneously the samples were prepared for the second experiment. A measure of 1 mL of bovine serum albumin (0.04 g/mL) was mixed with 10 µL of the previously prepared I, II, and III samples. Then, 1 mL of acetonitrile was added to the mixtures. Mixtures prepared in this way were vortexed for 20 s and set aside for 15 min at room temperature. Then the resulting suspension was applied onto the chromatographic plate and investigated.

### 3.3. Plate Preparation

Commercially available chromatographic plates were cut into smaller pieces (5 × 10 cm) with a TLC plate cutter (OM Laboratory, Chigasaki, Japan). The adsorbent layer of the plate was then washed by immersing in methanol for 1 min, dried in the air, and then activated at 105–110 °C for 15 min. Next, the plates were left in a desiccator for cooling.

### 3.4. Application of the Samples on the Chromatographic Plate

The samples were applied as a 2 µL volume droplet onto the adsorbent surface of the chromatographic plate using an automatic pipette Optipette from HTL (Warsaw, Poland).

### 3.5. Optimization of Conditions of the Substances Separation with TLC/HPTLC

Preliminary studies were performed to choose the conditions which allowed the best solute isolation. At this stage of the experiment, the chromatograms were developed in the horizontal thin-layer chromatography chamber DS 10 × 10 cm (Chromdes, Lublin, Poland) at a distance of 45 mm of the mobile phase migration. In the second series of experiments, the chromatograms of the samples were developed with the system containing impregnated sorbent. For this purpose, another chromatographic plate with an adsorbent layer impregnated with bovine serum albumin (0.04 g/mL water solution) was prepared. The 15 × 33 cm horizontal chamber prototype was used (Department of Physical Chemistry, Medical University, Lublin, Poland) for this process. Other details of the impregnation process were presented in our previous paper [18].

All experiments were performed in triplicate.

### 3.6. Stage of Sample Preparation

A prototype horizontal chamber with a moving pipette driven by 3D printer mechanism (Infinum 3D, Lublin, Poland) was used to develop the investigated solutes’ chromatograms with the controlled eluent flow velocity. During this process, the chromatographic plate was placed in a horizontal Teflon chamber with the adsorbent layer face-up. Then, the mobile phase was delivered onto the adsorbent layer using the moving pipette. A computer program controlled the movement of the pipette over the adsorbent layer surface.

**Figure 3:**
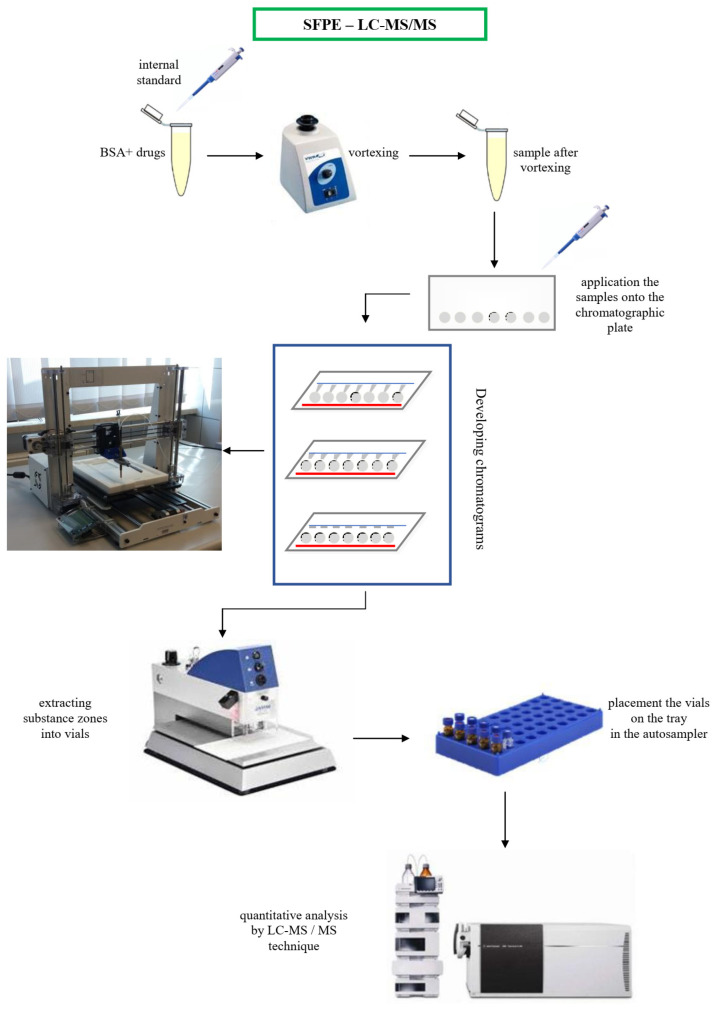
Graphical presentation of the procedure of biological sample analysis.

The details of the SFPE procedure using a prototype device for semiautomatic sample preparation were presented in [17]. The procedure of biological sample analysis is graphically presented in Figure 3.

Stage 1—the internal standard addition, vortexing, and application of the samples onto the chromatographic plate;

Stage 2—developing the chromatograms in the prototype horizontal chamber with a 3D printer mechanism. The method of delivering eluent to the mobile phase is shown in the video (the video is included in the Supplementary Materials).

Stage 3—extraction of substance zones (localized at the mobile phase front) into the vials.

Stage 4—LC–MS/MS quantification.

#### 3.6.1. Spot Narrowing Procedure

**Figure 4:**
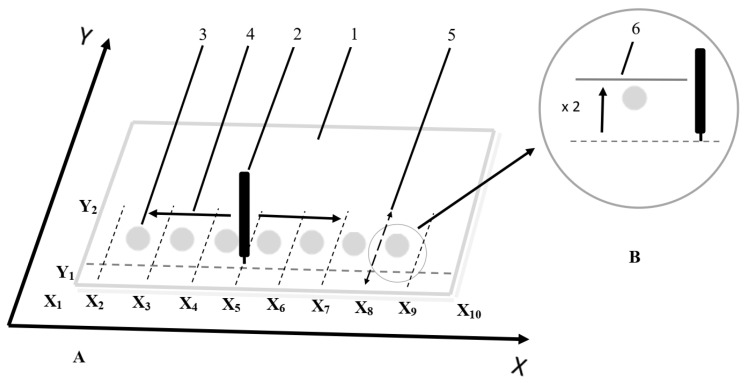
(**A**) Scheme of developing chromatograms with the use of moving pipette driven by a 3D machine. 1—chromatography plate, 2—moving pipette, 3—sample spots applied on the chromatographic plate; 4—the direction of movement of the pipette when developing chromatograms; 5—the direction of movement of the pipette when narrowing the starting spots. The arrows indicate the direction of movement of the pipette. (**B**) Wetting the starting spot twice before developing the chromatogram for the first time. 6—mobile phase front.

The narrowing of the solute zones was necessary to eliminate the non- homogenous solute dispersion that often originates from the radial chromatography effect during sample application onto an adsorbent layer of the TLC/HPTLC plate. The homogenous substance dispersion in the final spot at solvent front position is an indispensable condition for quantitative analysis of investigated compounds with the SFPE procedure. The procedure for this step of experiments was performed using the methanolic solutions of investigated compounds. Seven spots (zones) of the investigated compounds were formed onto the chromatographic plate (Figure 4A) with an automatic pipette. After evaporation of the solvent, the plate was placed in the device chamber, and the process of narrowing the zones of the substance was started. The pipette that delivered the mobile phase was moved with the use of the 3D machine along eight paths, each perpendicular to the X-axis at positions X2 … X9 located between the starting spots (Figure 4A). The length of each of the eight paths of the pipette movement was specified by the Y-axis in two positions, “Y1-Y2-Y1”, where Y1 = 5 mm and Y2 = 25 mm, measured from the edge of the plate. The pipette movement speed was set to 2000 mm/min, while the delivery rate of the mobile phase to the adsorbent layer was set to 6 mL/h. After the narrowing process, the chromatographic plate was dried under a fume hood for 10 min. The above-mentioned procedure forms the S part of the plate development.

#### 3.6.2. Planar Chromatogram Development

In each variant of the chromatographic plate development (part P of the plate development), the chromatograms were developed with a pipette parallelly moved toward the X-axis, while the position of pipette toward the Y-axis was constant (Y3 = 10 mm—as measured from the lower edge of the plate (Figure 4A)). The distance migration of the mobile phase was 35 mm measured from the start line. The pipette speed was set to 2000 mm/min, while the mobile phase delivery rate was 6 mL/h. The distance between the pipette tip and the surface of the adsorbent layer was 0.15 mm. Additionally, for the samples with bovine serum albumin, the spot was wetted twice with the mobile phase before the first development of the chromatograms. The process of wetting the zones is shown in Figure 4B.

The following variants of the SPFE procedure for the sample preparation before the quantification step were used:

SP2 variant—the chromatographic plate with applied spots (see Figure 4A) was placed in a prototype horizontal chamber with a 3D printer mechanism, and the chromatogram was developed once using the narrowing S procedure described above (in Section 3.6.1). Then the plate was dried under a fume hood for 10 min. Next, the P part of the development was performed. After each chromatogram development, the adsorbent layer of the plate was dried under a fume hood for 10 min. Each chromatogram was developed twice with the use of the P part of the procedure. Next, the solute/solute zones localized at the mobile phase front (final position of the mobile phase front) was/were extracted (isolated). This process was carried out with the use of the TLC–MS Interface from CAMAG (the mobile phase flow: 0.3 mL/min). 0.1% solution of formic acid in methanol (LC–MS grade) was used as the extractant. For quantification, the extracted sample solutions (10 µL) were injected into the HPLC column.

P3 variant—the chromatographic plate with applied spots (see Figure 4A) was placed in a prototype horizontal chamber with a 3D printer mechanism; before the first chromatogram development, the zone was wetted twice with the mobile phase (Figure 4B). Then the plate was evaporated under a fume hood for 10 min. Next, it was placed in the chamber, and the chromatogram was developed twice. After each development of the chromatogram, the plates were dried under a fume hood for 10 min. The remaining processes were identical to the SP2 variant.

Direct LC–MS variant (dLC–MS)—substances were determined by straightforward introduction into the LC–MS/MS system (Liquid Chromatography Tandem-Mass Spectrometry, Agilent, Santa Clara, CA, USA), without prior sample preparation by means of the SFPE procedure, i.e., the standard methanol solution without biological matrix.

### 3.7. Visualization of Substance Zones

The TLC Visualizer, CAMAG (Muttenz, Switzerland), was used for plate image documentation of the solute zones onto the adsorbent layer. Documentation was collected at a wavelength of 254 nm.

### 3.8. Instrumentation

The Agilent 1290 Infinity LC System(Santa Clara, CA, USA) connected with Agilent 6460 Triple Quadrupole was used for the LC–MS experiments. Column Zorbax Eclipse Plus-C18 column (4.6 × 100 mm, 3.5 µm, Agilent, Santa Clara, CA, USA) was applied. The mobile phase consisted of 50 % acetonitrile (containing 0.1% formic acid) and 50% water (containing 0.1% formic acid). The data were acquired at the positive ionization mode, with the exception of hydrochlorothiazide (negative mode). The capillary voltage was 2500 V. The nebulizer gas setting was 30 psi. The ion source was operated at the temperature of 300°C and the drying gas setting was 9 L/min. The sheath gas flow was 12 L/min, while the sheath gas temperature was 400°C.

## 4. Conclusions

This paper shows that the SFPE procedure of biological sample preparation (matrix of bovine serum albumin), performed with the prototype device, can be used for effective quantitation of the investigated antihypertensive drugs by means of the LC–MS technique. The results obtained from the SFPE procedure were reproducible and satisfactory. The %RSD in real samples did not exceed 6%; the relative error was below 10% for five of the seven drugs analyzed. Recovery of the majority of the investigated solutes at the level within 100% and low LOD and LOQ values additionally confirms the effectiveness of our procedure for the preparation of biological samples with antihypertensive drugs before their further quantitative determinations.

The use of a moving pipette driven by the 3D machine to develop chromatograms in the SFPE procedure significantly reduces the number of manual operations required compared to the classic unfolding method and enables further automation of the process.

The mobile pipette, which enables the delivery of the mobile phase to any position on the chromatographic plate surface, allows the development of chromatograms in two directions. Because of this, we can prepare several samples at the same time, which shortens the analysis time and reduces solvent consumption.

Further investigations are in the pipeline, and we hope that the obtained results will confirm the usefulness of the SFPE procedure in monitoring the concentration of antihypertensive drugs in the patient’s blood.

## Figures and Tables

**Table 1:** Selected analytical parameters characterize SPE and precipitation [6,7,8,9,10,12,13,14]. The calculations were carried out for the protein precipitation procedure involving 24 samples (wherein 24 samples were simultaneously centrifuged) and 96 samples in the case of the SPE procedure (maximum size of the SPE vacuum manifold).

Analytical Parameter	SPE	Precipitation
Time per sample (min)	1.5–3	2–5
Average consumption of solvent (mL) per sample	2.5–8	0.6–1.5
Number of steps (operations)	8–12	6–7
The cost of the procedure calculated regarding solvent consumption (EUR)	0.45–1.25	0.15–0.35
Necessary equipment	SPE vacuum manifold; solid phase extraction columns	fridge; special centrifuge for biological samples
Automation	Partial	No

**Table 2:** The R_f_ values of the test substances and the internal standards in different chromatographic systems.

Ramipril						
	Acetone	Acetonitrile	Ethyl Acetate	Isopropanol	Toluene	Methanol
HPTLC Silica gel 60 F_254_ HPTLC RP-18 F_254_ HPTLC DIOL F_254_ HPTLC CN F_254_ TLC Cellulose F	0.69 *	0	0.34	0.51 *	0	0.92
	0.92	0.59 *	0.88 *	0.78	0	0.78
	0	0	0.35	0.66	0	0.92
	0.94	0.71	0.69	0.68	0	0.89
	0.98	0.96	0	0.91	0	0.96
**Lercanidipine**						
HPTLC Silica gel 60 F_254_ HPTLC RP-18 F_254_ HPTLC DIOL F_254_ HPTLC CN F_254_ TLC Cellulose F	0.93	0.51	0.92	0.92	0	0.96
	0.94 *	0	0	0.53 *	0	0.15
	0	0	0	0.55	0	0.95 *
	0	0	0	0	0	0
	0	0	0	0.89 *	0	0.94 *
**Indapamide**						
HPTLC Silica gel 60 F_254_ HPTLC RP-18 F_254_ HPTLC DIOL F_254_ HPTLC CN F_254_ TLC Cellulose F	0.93	0.94	0.92	0.95	0	0.98
	0.98	0.96	0.95	0.98	0	0.92
	0.98	0.92	0.92	0.95	0	0.95
	0.98	0.98	0.94	0.75	0	0.88
	0	0	0	0.89 *	0	0.94 *
**Valsartan**						
HPTLC Silica gel 60 F_254_ HPTLC RP-18 F_254_ HPTLC DIOL F_254_ HPTLC CN F_254_ TLC Cellulose F	0	0	0	0.22	0	0.99
	0.98	0.93	0.98	0.97	0	0.95
	0.96	0.88	0.91	0.98	0	0.98
	0.94	0.98	0.98	0.92	0	0.94
	0	0	0	0	0	0.95
**Hydrochlorothiazide**						
HPTLC Silica gel 60 F_254_ HPTLC RP-18 F_254_ HPTLC DIOL F_254_ HPTLC CN F_254_ TLC Cellulose F	0.94	0.94	0.64	0.92	0	0.97
	0.98	0.97	0.87	0.96	0	0.96
	0.96	0.91	0.79	0.91	0	0.91
	0.94	0.98	0.91	0.59	0	0.83
	0	0	0	0	0	0.68
**Telmisatran**						
HPTLC Silica gel 60 F_254_ HPTLC RP-18 F_254_ HPTLC DIOL F_254_ HPTLC CN F_254_ TLC Cellulose F	0.35 *	0	0.12	0.44	0	0.98
	0.56 *	0	0	0.45	0	0.75
	0.38	0	0.15	0.41	0	0.60
	0.32	0	0.15	0.25	0	0.73
	0.98 *	0	0	0	0	0.95
**Perindopril**						
HPTLC Silica gel 60 F_254_ HPTLC RP-18 F_254_ HPTLC DIOL F_254_ HPTLC CN F_254_ TLC Cellulose F	0	0	0.26	0.41	0	0.78
	0.93	0.56	0.84	0.77	0	0.73
	0	0	0	0.64	0	0.92
	0.91	0.69	0.64	0.67	0	0.88
	0.98	0	0	0.93	0	0.88
**Nebivolol**						
HPTLC Silica gel 60 F_254_ HPTLC RP-18 F_254_ HPTLC DIOL F_254_ HPTLC CN F_254_ TLC Cellulose F	0.11	0	0	0.33 *	0	0.41
	0.92 *	0	0	0.73	0	0.84
	0	0	0	0.61	0	0.96
	0.38	0.22	0.16	0.44	0	0.15
	0	0	0	0	0	0.83

* tailing the substance zone.

**Table 3:** ^n^R_f_ of solutes vs. the number of developments. Chromatographic plate HPTLC silica gel 60 F_254_.

Substance	0.1% Formic Acid in Methanol		0.1% Ammonia in Methanol	
	Single Development	Two Developments	Single Development	Two Developments
Perindopril	0.88	0.99	0.83	0.97
Ramipril	0.91	0.99	0.90	0.99
Lercanidipine	0.92	0.99	0.94	0.99
Indapamide	0.96	0.99	0.96	0.99

**Table 4:** The values of statistical parameters obtained for methanolic samples, calculated on the basis of the average (*n* = 7). Analysis followed the extraction from final solvent front position by methanol with 0.1% formic acid with TLC–MS Interface (SFPE procedure), respectively. Stationary phases, chromatographic plate HPTLC silica gel 60 F_254_; HPTLC diol F_254_. Applied symbol explanations: %RSD is the standard deviation; %RE is the relative error defined as 100(Mx-dLC–MS)/dLC–MS, where Mx-mean is the value of the peak area ratio substance/internal standard obtained with the use of LC–MS/MS method after sample preparation using the SFPE technique; dLC–MS is the mean value of the substance/internal standard peak area ratio obtained with the use of LC–MS/MS method without the use of the SFPE technique. The solute recovery is calculated from the formula Mx/dLC–MS × 100. The limit of quantitation (LOQ) and the limit of detection (LOD) was calculated using the formulae: LOD = 3.3σ/s and LOQ = 10σ/s, respectively, where σ is the standard deviation of the response, s is the regression line slope.

I Group of Substances *					
	%RSD	%RE	%Recovery	LOD (µg/L)	LOQ (µg/L)
Lercanidipine	2.89	2.04	97.96	2.17	6.60
Ramipril	4.32	1.52	98.47	3.95	11.89
Indapamide	5.91	0.97	99.03	2.54	8.08
II Group of Substances *					
	**%RSD**	**%RE**	**%Recovery**	**LOD (µg/L)**	**LOQ (µg/L)**
Valsartan	1.25	0.55	99.44	2.57	7.63
Hydrochlorothiazide	3.52	3.33	96.67	0.85	2.58
III Group of Substances **					
	**%RSD**	**%RE**	**%Recovery**	**LOD (µg/L)**	**LOD (µg/L)**
Perindopril	1.20	5.24	94.76	2.90	8.79
Nebivolol	2.59	0.95	100.95	2.28	6.93

* HPTLC silica gel 60 F_254_; ** HPTLC diol F_254_.

**Table 5:** The values of statistical parameters obtained for bovine serum albumin samples and bovine serum albumin sample with acetonitrile, calculated on the basis of the average (*n* = 7). Analysis followed the extraction from final solvent front position by methanol with 0.1% formic acid with TLC–MS Interface (SFPE procedure), respectively. Stationary phase chromatographic plate HPTLC silica gel 60 F_254_; HPTLC diol F_254_.

Bovine Serum Albumin Sample						Bovine Serum Albumin Sample with ACN				
I Group of Substances *										
	%RSD	%RE	%Recovery	LOD (µg/L)	LOQ (µg/L)	%RSD	%RE	%Recovery	LOD (µg/L)	LOQ (µg/L)
Lercanidipine	6.31	16.47	83.53	4.06	12.3	4.04	8.33	91.67	1.19	3.61
Ramipril	6.48	18.31	81.69	2.31	7.02	5.31	6.71	93.29	1.3	3.93
Indapamide	18.50	90.44	9.56	8.08	24.49	5.64	80.11	19.89	8.53	25.8
II Group of Substances *										
	**%RSD**	**%RE**	**%Recovery**	**LOD** **(µg/L)**	**LOQ** **(µg/L)**	**%RSD**	**%RE**	**%Recovery**	**LOD** **(µg/L)**	**LOQ** **(µg/L)**
Valsartan	5.97	3.48	103.48	3.18	9.65	5.40	2.39	102.39	2.78	8.42
Hydrochlorothiazide	4.81	20.57	79.43	6.38	19.34	5.76	13.28	86.72	6.41	19.42
III Group of Substances **										
	**%RSD**	**%RE**	**%Recovery**	**LOD** **(µg/L)**	**LOQ** **(µg/L)**	**%RSD**	**%RE**	**%Recovery**	**LOD** **(µg/L)**	**LOQ** **(µg/L)**
Perindopril	2.66	2.07	102.07	2.31	7.02	2.65	1.95	98.05	1.19	3.61
Nebivolol	5.29	19.49	80.51	2.72	8.26	5.18	9.80	90.2	3.25	9.84

* HPTLC silica gel 60 F_254_; ** HPTLC diol F_254._

**Table 6:** Analysis of SFPE procedure. Stationary phase: chromatographic plate HPTLC silica gel 60 F_254_. Mobile phase: methanol.

Chromatographic plate dimensions	5 × 10 cm	10 × 20 cm
Time per sample (min)	8.20	2.40
Average consumption of solvents (mL) per sample	0.41	0.38

**Table 7:** The analytical parameters characterizing the SFPE procedure. The calculations were carried out for 32 samples (limitation being due to the size of the chromatographic plate).

Analytical Parameter	SFPE
Time per sample (min)	2.40
Average consumption of solvents (mL) per sample	0.38
Number of steps (operations)	6
The cost of the procedure calculated regarding solvent consumption (EUR)	0.35
Necessary equipment	Horizontal chamber with pipette driven by 3D printer mechanism; TLC–MS Interface
Automation	Partial

## Supplementary Materials

The following are available online, Video S1: The method of delivering eluent to the mobile phase.

## Sample Availability

Samples of the hypertensive drugs are available from the authors.
